# Declining growth of natural history collections fails future generations

**DOI:** 10.1371/journal.pbio.3001613

**Published:** 2022-04-19

**Authors:** Vanya G. Rohwer, Yasha Rohwer, Casey B. Dillman

**Affiliations:** 1 Cornell University Museum of Vertebrates, Department of Ecology and Evolutionary Biology, Ithaca, New York, United States of America; 2 Oregon Institute of Technology, Klamath Falls, Oregon, United States of America

## Abstract

The growth of natural history collections is declining. We need to reverse this decline to effectively facilitate the discovery of new knowledge and inform future societies about their past.

## Teaser

New additions of vertebrate specimens to natural history collections are declining in the midst of widespread and accelerating environmental change. Reversing these trends in collecting efforts is essential for addressing future unforeseen ecological issues.

When the explorer and ornithologist Rollo Beck collected a series of seabird specimens in 1907, he never would have imaged that, a century later, researchers would rely on his specimens to answer critical questions about ecosystem change [1,2]. Who could have imagined that peregrine falcon egg sets would reveal the harmful effects of the insecticide DDT [3], that amphibian specimens from throughout the Americas would inform our understanding of virulent forms of chytrid fungus [4], or that bird and mammal collections would illuminate unanticipated consequences of climate change [5]? Natural history collections help solve conservation problems and answer unforeseen questions that simply could not have been asked about natural systems when those specimens were collected.

**Fig 1:**
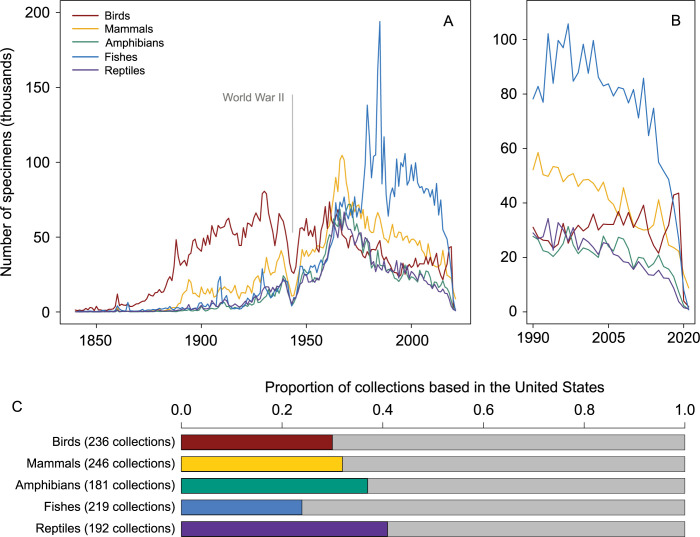
Growth of natural history collections through time using the subset of the world’s collections available through GBIF. **(A)** Broad fluctuations exist across 5 vertebrate groups in the number of specimens added to natural history museums since 1840. **(B)** Recent declines in collections growth from 1990 to 2021. **(C)** GBIF includes collections from over 60 countries, but many collections are based in the United States of America. Colored bars indicate the proportion of USA-based collections for each vertebrate group, which represent 25% to 42% of data. GBIF, Global Biodiversity Information Facility.

Despite the benefits of natural history collections, fewer and fewer specimens are being added to them. Data from over 245 institutions, available through the Global Biodiversity Information Facility (GBIF), show that the addition of new physical specimens has declined by 54% to 76% across 4 vertebrate groups from 1965 to 2018 (Fig 1); fish are the exception for this time period, having declined by only approximately 1% because of a pronounced spike in collecting activity in 1985. Between 1990 and 2019, collecting activity has been more idiosyncratic across taxa, with the addition of new specimens increasing slightly for birds and dropping sharply for all other taxa. For several vertebrate groups, collecting activity is declining so precipitously that it is now lower than it was during World War II.

These declines are alarming. Failing to add new specimens to natural history collections compromises the value of existing collections and, inevitably, will limit future discovery through a lack of appropriate comparative material. If we value the inspiration, insight, and use of specimens in the discovery of new knowledge, we should quickly reverse these trends.

Many of the factors that contribute to declining collections growth fall into 2 categories: institutional and ethical. Several university-based collections have become less active as the questions that initially fueled the growth of collections are now viewed as dated or addressable using nonlethal samples. Consequently, teaching and research programs shifted away from using specimens, resulting in collections being undervalued, unused or, worse, closed. Parallel declines in growth rates of plant [6] and insect [7] collections likely reflect similar changes in fashionable research programs and not increased ethical controversy surrounding the collection of these taxa. For vertebrates, however, the recognition that animals are sentient creatures with their own interests makes scientific collecting ethically more controversial now than it was in the past.

Collecting specimens pits 2 valuable things against each other: the life of a sentient animal versus the immediate knowledge gained by taking that animal’s life. The focus on immediate knowledge fails to address the unforeseen importance of specimen data for answering questions that were not, or could not have been, asked when the specimen was collected. In short, this argument ignores the unknowable future value of specimens [8]. Debates over the ethics of collecting often focus on the killing of individuals without thoughtful consideration of what will be lost if we cease collecting. Will existing collections be sufficient for future scientific studies? How will an absence of contemporary physical specimens undermine future conservation efforts? What new discoveries will future technologies reveal from using old specimens? Unfortunately, there are no easy answers and no escaping the ethical tension tied to scientific collecting.

Current declines in collecting activity also come amid the most dramatic environmental changes to have occurred since the Enlightenment. Documenting and responding to these changes frequently relies on retrospective analyses that involve the application of new technologies to specimens that were collected before environmental changes occurred. Uncertainties in both the questions we will ask and the technologies we will have in the future highlight 2 important points: collecting organisms, as opposed to nonlethal tissue samples, maximizes data for future studies, and specimens have an irreplaceable role in understanding unforeseen changes in natural systems.

Declines in the number of physical specimens collected also coincide with dramatic increases in observational data. The notion that observational specimens or nonlethal samples (such as blood, fur samples, fin clips, or photographs) are comprehensive replacements for traditional specimens is mistaken. These data are narrower in focus, limiting their potential to be used to address questions beyond the scope for which they were originally collected. We recognize that different specimen types—physical and observational—have their strengths and weakness, and we see opportunities to better understand populations by combining these data sets. However, nonlethal sampling approaches are not comprehensive replacements for physical specimens.

The decline in collecting is also ethically problematic. Because scientific collecting is typically justified by the knowledge gained from specimens, this ethical justification should also extend to existing specimens and to the knowledge that is lost by failing to collect new specimens. Declines in collecting create unfillable gaps in one of our longest running data sets about natural systems, which erodes the value of existing collections for future discovery and compromises the ethical justification for both current and past collecting.

The question that remains to be addressed is whether this trend can be reversed. Growth of collections can come in many ways. University-based museums that train future curators should have active collecting programs that teach researchers how to use collections and how active research programs can complement and guide the growth of collections. Growing nontraditional collections of specimen attributes (such as extended wings and syrinxes in birds, nests, or specimens at different developmental stages) provides novel specimen types while building scientifically useful collections. For some taxa, developing coordinated salvage networks across museums, government agencies, nongovernmental organizations, and other relevant parties offer promising ways to build collections, especially of species only obtainable through salvage because of rarity. Developing capacity locally and abroad will deepen our understanding of less studied taxa and areas, and these discoveries that should be led by local researchers.

We also have to continue the ethical conversation. We should not dismiss the concerns of individuals about collecting, but we need to be clear about the knowledge gained from natural history collections and how this knowledge is essential to so many scientific disciplines. If we, as a scientific community, value understanding changes in populations and ecosystems through time and the accumulation of scientific knowledge, then scientific collecting should continue. If the declines in collecting continue, the legacy we leave future generations will be an unfillable gap in the longest tangible record of life on earth, created precisely in the midst of widespread and dramatic environmental change. Is this a risk we are willing to take?

## References

[1] Beissinger SR, Perry MZ. Reconstructing the historic demography of an endangered seabird. Ecology. 2007;88:296–305. doi: 10.1890/06-0869 17479748

[2] Becker BH, Beissinger SR. Centennial decline in the trophic level of an endangered seabird after fisheries decline. Conserv Biol. 2006;20:470–9. doi: 10.1111/j.1523-1739.2006.00379.x 16903108

[3] Ratcliffe DA. Decrease in eggshell weight in certain birds of prey. Nature. 1967;215:208–10. doi: 10.1038/215208a0 6049131

[4] Rodriguez D, Becker CG, Pupin NC, Haddad CFB, Zamudio KR. Long-term endemism of two highly divergent lineages of the amphibian-killing fungus in the Atlantic Forest of Brazil. Mol Ecol. 2014;23:774–87. doi: 10.1111/mec.12615 24471406

[5] Riddell EA, Iknayan KJ, Hargrove LH, Tremor S, Patton JL, et al. Exposure to climate change drives stability or collapse of desert mammal and bird communities. Science. 2021;371:633–6. doi: 10.1126/science.abd4605 33542137

[6] Prather LA, Alvares-Fuentes O, Mayfield MH, Ferguson CJ. The decline of plant collecting in the United States: A threat to the infrastructure of biodiversity studies. Syst Bot. 2004;29:15–28.

[7] Fischer EE, Cobb NS, Kawahara AY, Zaspel JM, Cognato AI. Decline of amateur Lepidoptera collectors threatens the future of specimen-based research. Bioscience. 2020;71:396–404.

[8] Remsen JV. The importance of continued collecting of bird specimens to ornithology and bird conservation. Bird Conserv Int. 1995;5:146–80.

